# Ultra-Violet Electroluminescence of ZnO Nanorods/MEH-PPV Heterojunctions by Optimizing Their Thickness and Using AZO as a Transparent Conductive Electrode

**DOI:** 10.3390/ma12182976

**Published:** 2019-09-14

**Authors:** S. Wageh, Su-Ling Zhao, Xiao-Yi Xie, Song Gao, Ahmed Al-Ghamdi

**Affiliations:** 1 Department of Physics, Faculty of Science, King Abdulaziz University, Jeddah 21589, Saudi Arabia; aghamdi90@hotmail.com; 2 Physics and Engineering Mathematics Department, Faculty of Electronic Engineering, Menoufia University, Menouf 32952, Egypt; 3 Institute of Optoelectronic Technology, Beijing Jiaotong University, Beijing 100044, China; 12121654@bjtu.edu.cn (X.-Y.X.); 11121777@bjtu.edu.cn (S.G.)

**Keywords:** electroluminescence, ZnO nanorods, heterojunctions

## Abstract

In this paper, a series of ITO/ZnO/ZnO nanorods/MEH-PPV/Al were prepared with different thicknesses of MEH-PPV that were changed from 15, 10 to 7 nm. The electric field in the devices was analyzed. An increase in the electric field on ZnO made hole injection easy and the electrons tunnel fast through thinner MEH-PPV to ZnO. This made the carriers prefer to recombine inside the ZnO layer, and the emission of ZnO was predominant under direct current (DC) bias. Furthermore, another device was fabricated with the structure of AZO (Al-doped ZnO)/ZnO/ZnO nanorods/MEH-PPV/Al. Ultra-violet (UV) electroluminescence (EL) at 387 nm from ZnO band edge emission was realized under DC bias. The turn-on voltage of the devices having AZO as the electrode is lower than that of ITO, and the EL power is enhanced. This work also studies the effect of inserting LiF underneath the Al electrode and above the layer of MEH-PPV. The LiF film inserted caused an obvious decrease in turn-on voltage of the devices and a pronounced increase in the EL power. The mechanism of electroluminescence enhancement is also discussed.

## 1. Introduction

Oxide semiconductors with a wide direct band gap and exciton with large binding energy like zinc oxide have various optoelectronic applications. For several years, ZnO luminescence has been extensively studied due to its important optoelectronic applications. It is used in laser operating devices and light emitting devices emitted in the ultraviolet region [1,2,3,4] ZnO is seen as a promising compound that can be used for light emission in ultra-violet (UV) region technology [5] in the next generation. However, there is a problem in carrying out research on ZnO successfully. This is the difficulty of fabricating p-type material of ZnO, apart from the fact that the wide band gap structures usually establish a feeble efficiency of p-type doping [6]. As a result, researchers now use different aspects to build ultraviolet light emitting devices. Several studies have been done to build light emitting devices using organic materials and tried to achieve the emission. However, these devices are not stable. One of the ways to solve this stability problem of these devices built with pure organic materials is to use hybrid structure of inorganic and organic materials. Thus, a heterostructure consisting of an ZnO n-type semiconductor that works as an active layer and organic material that works as p-type compounds has been reported. P-type polymers such as poly (3,4-ethylenedioxythiophene) poly(styrenesulfonate) (PEDOT:PSS), poly(9-vinylcarbazole) (PVK), 4,4′-bis(N-carbazolyl)-1,1′-biphenyl (CBP), poly(9, 9-dioctylfluorene) (PFO) [7,8,9,10,11], and poly[2-methoxy-5-(20-ethylhexoxy)-1,4-phenylenevinylene] (MEH-PPV) have been used in this heterostructure to prepare light emitting diodes (LEDs) [7,8,9,10,11,12]. However, the electroluminescence in these devices were the exciton emission of organic materials or the defect emission of ZnO [9,10,11,12]. The UV band-edge electroluminescence of ZnO were very weak comparatively and not adequate [7,8]. Another structure with UV electroluminescence has been produced. It consists of a ZnO nanorods/MEH-PPV heterostructure in which a MEH-PPV polymer is applied as the electron injection layer. This structure nearly shows ultraviolet electroluminescence of ZnO at 380 nm under DC bias [13] but has very weak intensity of N-UV. To enhance N-UV electroluminescence intensity, indium tin oxides (ITO) substrates and ZnO nanorods were treated with oxygen-plasma. This treatment increased their work function and lowered sheet resistance along with smooth surface [14]. The device treated showed lower turn-on voltage and increased EL power compared to the devices that were not treated with oxygen-plasma. The EL power obtained is just about 70 nW, which is still not high. Another way to improve the N-UV electroluminescence power of ZnO Nanorods/MEH-PPV heterostructure devices is to replace ITO with a new kind of electrode (TCO).

The new kind of transparent conductive oxide (TCO) layer is AZO (Al-doped ZnO), which has many advantages. It is rich in raw materials, is environmentally friendly, and has a high work function [15]. However, proper doping of Al does not change the crystal structure of ZnO [16]. Due to the higher work function and lattice matching of AZO, it is more suitable as the electrode in ZnO nanorods/MEH-PPV heterostructure devices. Thus, we fabricated different devices with these two kinds of electrodes, and discussed their luminescence mechanism.

## 2. Materials and Methods 

The devices were fabricated as follows. The glass substrates coated with ITO and AZO were cleaned in an ultrasonic path using deionized water, ethanol, and acetone, and dried with nitrogen gas, respectively. Then, the hydrothermal method with a two-step was used to grow ZnO nanorods on ITO and AZO glass substrates at low temperatures. To facilitate the nucleation and growth of ZnO nanorods, the zinc acetate dehydrate solution was spin-coated on top of ITO and AZO glass substrates firstly. Then, they were annealed in ambient atmosphere at 400 °C for 30 min to form a seeding layer of ZnO. The followed second step of hydrothermal reaction for growth of ZnO nanorods was proceeded by mixing 25 mM hexamethylenetriamine (C_6_H_12_N_4_) with 25 mM of (Zn(NO_3_)_2_·6H_2_O in 200 mL of deionized water in a glass flask. The substrates with ZnO seed layer were immersed in the mixture solution and maintained at 92 °C for 15 min in an electric thermostatic heated water bath. Then, these substrate samples were taken out of the solution and washed with ethyl alcohol and deionized water sequentially. The obtained samples were annealed in ambient atmosphere at 400 °C for 30 min to crystalize ZnO nanorods layer. An insulator polymer Polymethylmethacrylate (PMMA) dissolved in chloroform with a concentration of 5 mg/mL was spin-coated onto the ZnO nanorods films with a rotation rate of 3000 rpm to fill the interspace between nanorods and insulate each other. Then, a poly [2-methoxy-5-(2-ethylhexyloxy)-1, 4-phenylene vinylene] (MEH-PPV) p-type polymer was dispersed in chloroform with a concentration of 5 mg/ml, and spin-coated on the as-grown ZnO nanorods layers. Three samples were fabricated with different spin-coated rates of rotation with 2500, 3000 and 3500 rpm; they produced MEH-PPV films with thicknesses of 7, 10, and 15 nm, respectively. Then, the fabricated samples were annealed in a vacuum drying oven at 80 °C for 15 min. Finally, a 100 nm thick Al film was thermally evaporated under a high vacuum condition of 2 × 10^–4^ Pa. The devices fabricated with various thicknesses are given symbolic names. The names of the devices having MEH-PPV thicknesses of 7,10 and 15 are called AI, AII, and AIII, respectively. Another device was fabricated to investigate the effect of replacing ITO with AZO electrode (hereafter called device B). Furthermore, to decrease the turn-on voltage, another device was fabricated with deposition of 0.8 nm LiF film on top of MEH-PPV layers and underneath Al film by thermal evaporation (hereafter called device C). In all devices, polymethyl methacrylate (PMMA) was used only to fill the interspace of nanorods to improve the device stability, so it is not discussed later about it. Finally, five devices were fabricated with different structures as follows:

Device AI: Al/(7 nm) MEH-PPV/ZnO nanorods/ZnO/ITO,
Device AII: Al/(10 nm) MEH-PPV/ZnO nanorods/ZnO/ITO,
Device AIII: Al/(15 nm) MEH-PPV/ZnO nanorods/ZnO/ITO,
Device B: Al/(10 nm) MEH-PPV/ZnO nanorods/ZnO/AZO,
Device C: Al/LiF (0.8nm)/(10 nm) MEH-PPV/ZnO nanorods/ZnO/ITO.

**Figure 1:**
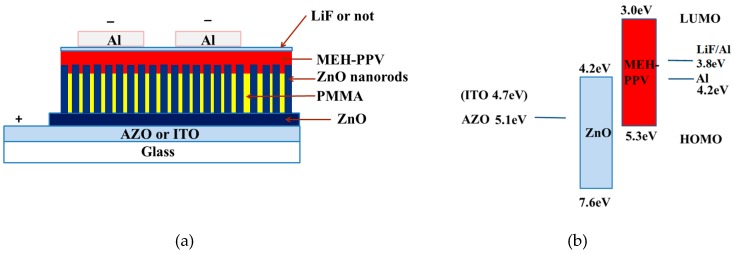
(**a**) schematic diagram and (**b**) energy band diagram of all the devices.

The schematic design of the devices and band energy diagram structure under forward bias is depicted in Figure 1. All devices have the same light area as 3 × 3 mm^2^. The forward bias on the devices means that the Al is working as cathode AZO or ITO is working as an anode. The device structure of Device AI, AII and AIII is the same as Al/MEH-PPV/ZnO nanorods/ZnO/ITO except for the different thickness of MEH-PPV. In order to compare the effect of substrates, the substrate of Device AII changed to AZO and Device B was prepared. A thinner layer LiF was added to improve the electron injection in Device AII and then Device C was fabricated. The devices were tested by measuring the EL spectrum under different forward bias conditions. An arrangement of Keithley 2410 (Tektronix, Inc., Beaverton, OR, USA) and a Newport 1830-C optical power meter mainframe (Newport Corporation, CA, USA) controlled by a computer and a 150 CCD spectrometer (Acton Research, ACTON, MA, USA) were used to investigate the EL and current–voltage–light power (I–V–L). All of these measurements were conducted at room temperature in an ambient atmosphere.

## 3. Results

**Figure 2:**
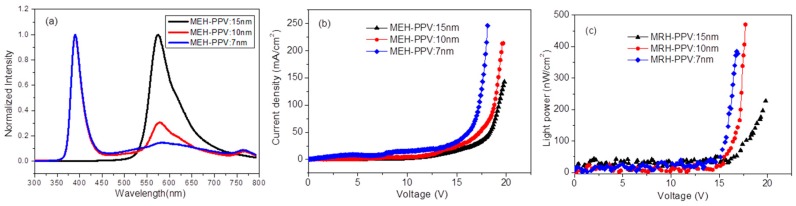
Electroluminescence spectra (**a**); J–V characteristics (**b**); and L–V characteristics (**c**) of three Device As with different MEH-PPV thicknesses.

**Figure 3:**
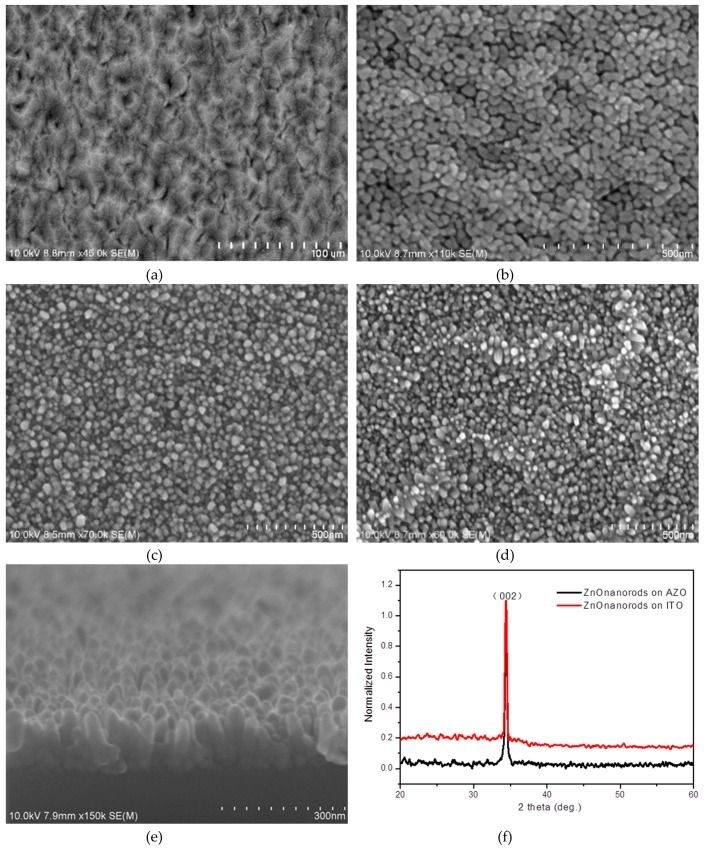
Top-view SEM of AZO surface (**a**), ZnO seed layer on AZO (**b**) and ZnO nanorods on AZO (**c**) and ITO (**d**), the cross-view SEM of ZnO nanorods on AZO (**e**), and XRD of ZnO nanorods (**f**).

**Figure 4:**
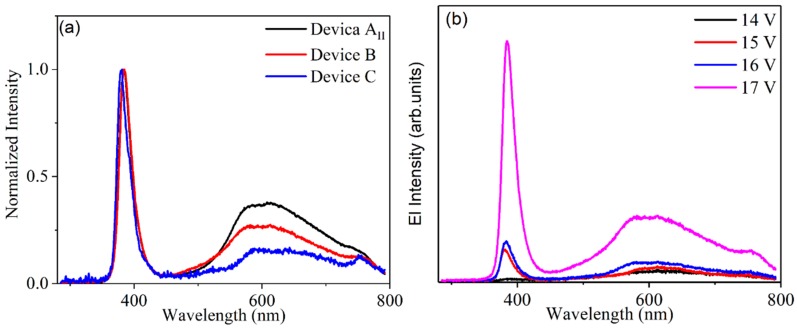
EL spectra of the three devices (**a**) and Device B under different biases (**b**).

**Figure 5:**
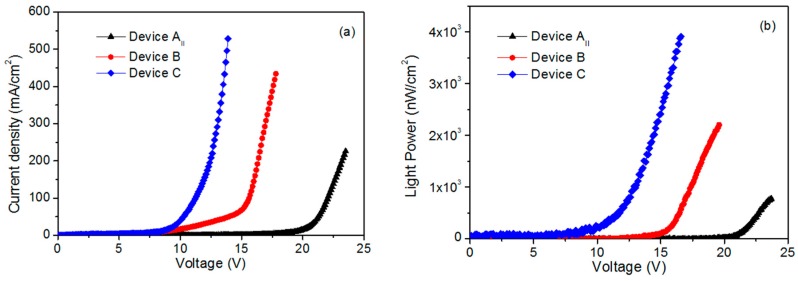
J–V characteristics (**a**) and L–V characteristics (**b**) of the three devices.

Figure 2a shows the electroluminescence spectrum of the devices with various thicknesses of MEH-PPV. The peak at 387 nm corresponded to the near band edge luminescence of ZnO and the peak around 575 nm originated from MEH-PPV bound electron–hole pair emission. Other emissions in the long wavelength came from the defect levels of ZnO. Figure 2b,c show the current density and light power of the devices with different MEH-PPV thicknesses of 15, 10 and 7 nm, respectively. Figure 3 shows the SEM views and XRD of ZnO nanorods on AZO and ITO. It is clear that ZnO nanorods on AZO have a uniform morphology. This is due to the good crystallization of ZnO seeds on AZO even though it has poor surface morphology. Figure 4 shows the normalized EL spectra and J–I–V characteristics of devices AII, B, and C with MEH-PPV thickness of 10 nm under forward bias. Figure 5a,b shows the I–V measurements and EL power of the three devices with different substrates and LiF layers.

All figures and tables should be cited in the main text as Figure 1 and Figure 2, etc.

## 4. Discussion

In previous studies, ultra-violet emission of ZnO was realized using the heterostructure of ZnO nanorods/organic materials (MEH-PPV, AlQ_3_, or PMMA). It is very important to control the carrier recombination zone to enhance the emission of ZnO. However, as the carrier is mobile, the mobility of the hole in ZnO is much greater than that of the organic materials. Thus, it is very easy to move the recombination zone into the organic layer or pass it to the opposite electrode as the leakage current. In order to let carriers recombine in ZnO, we applied the MEH-PPV organic layer with different thicknesses by varying the rate of spin-coating. It is very important to optimize the thickness of the organic layer in the heterostructure to balance carriers with a ZnO layer. Therefore, we analyzed the distribution of the electric field in the devices by changing the thickness of the organic layer. In our previous detection, the actual thickness of MEH-PPV on ZnO nanorods was only several nanometers. The distribution of the electric field on MEH-PPV and ZnO is analyzed according to their dielectric constants, respectively as follows:

(1) $$\frac{\varepsilon_{2}d_{1}}{\varepsilon_{1}d_{2}+\varepsilon_{2}d_{1}}V_{total}=V\left(MEH-PPV\right)$$

(2) $$\frac{\varepsilon_{1}d_{2}}{\varepsilon_{1}d_{2}+\varepsilon_{2}d_{1}}V_{total}=V\left(ZnO\right)$$

Where, *V*_total_ is the voltage on the whole devices where the effect of electrodes is ignored due to their high conductivity compared to ZnO and MEH-PPV. *ε*_1_ is the dielectric constant of MEH-PPV and equals 2.5, and *ε*_2_ of ZnO is equal to 10.3. The thickness of ZnO *d*_2_ is about 180 nm according to Figure 3e, and that of MEH-PPV on top of nanorods changes from 15, 10 to 7 nm. This makes the electric field on ZnO increase plus the injection current (Figure 2b). In addition, the thinner the layer of MEH-PPV is, the easier it would be to inject the carriers or tunnel through MEH-PPV to ZnO. Therefore, carriers will prefer to recombine inside the ZnO layer. 

It shows that the electroluminescence of ZnO and MEH-PPV is competitive (Figure 2a). When MEH-PPV is thicker, the electroluminescence from MEH-PPV is predominant and stronger than that of ZnO. This means that the carrier recombination zone is concentrated in the layer of MEH-PPV. Even though the voltage applied on MEH-PPV increases along with increased thickness of MEH-PPV, the inside electric field decreases under the same DC bias. Consequently, the electron injected and transported to MEH-PPV became poor. However, holes are weakly affected; they enter MEH-PPV to recombine with electrons in order to emit MEH-PPV. Contrarily, with decreased MEH-PPV’s thickness, the emission of ZnO will be easy. 

However, the emission of ZnO depends greatly on the morphology and crystallization of ZnO. In order to promote the quality of ZnO, we used another kind of substrate apart from ITO to prepare ZnO nanorods on them. AZO (Al-doped ZnO) as a transparent conductive oxide (TCO) layer is expected to realize the efficient electroluminescence of ZnO due to its improved interface quality resulting from the matched lattice between ZnO nanorods and AZO substrates. In addition, the work function of AZO is 5.1 eV, larger than that of ITO. Therefore, the hole injected from AZO to ZnO nanorods is easier than that from ITO to ZnO nanorods. 

It is clear that ZnO nanorods on AZO have a uniform morphology due to the good crystallization of ZnO seeds on AZO even though it has a poor surface morphology (Figure 3). ZnO has a similar lattice to AZO, which improves the crystallization of ZnO seeds as a buffer layer to prepare ZnO nanorods. On ITO substrates, the surface of the ZnO nanorod layer has a poor roughness. The normalized XRD pattern of ZnO nanorods in Figure 3f shows that ZnO nanorods are perpendicular to two kinds of substrates, respectively. The nanorods on AZO have a little better crystallization than on ITO because of the sharper peak (002) on AZO than on ITO. In Figure 4a, the electroluminescence was detected around 387 nm and 575 nm for ZnO and MEH-PPV, respectively. The determined turn-on voltage of light emitting device in which ITO is the electrode (device AII) is relatively high, ~20 V. When AZO is used as the electrode (device B), the turn-on voltage decreases to 15 V, which is smaller than that of the device AII. The device B emits light at 387 nm. This is predominantly under this lower voltage as shown in Figure 4b. As the bias increased, the defect emission of ZnO and the exciton emission of MEH-PPV were detected. It is expected to have an accumulation of holes in the ZnO nanorods layer under low bias. This is due to the high barrier of energy, which is nearly equal to 2.3 eV between the valence band of ZnO and the highest occupied molecular orbit of MEH-PPV. The high bias will support the injection of holes to MEH-PPV from ZnO. This will lead to a large percentage of recombination transfer from ZnO to MEH-PPV or to the interface between the two layers. For device AII, under a low bias, a large percentage of recombination originated from MEH-PPV. These results indicate that the defects of ZnO in device AII are more than those of device B. More defects in ZnO layer will trap the injected carriers. In such circumstances, the emissions arising from the ZnO defects and MEH-PPV exciton are easily observed in device AII under low voltage. With increased bias voltage, a large number of carriers will be injected into the conduction band and the valence band of ZnO, in order to enhance the emission at 387 nm. By replacing ITO with AZO, the defects of ZnO nanorods are lowered. Thus, the number of carriers trapped by defects are few under low voltage. Under lower voltage, the band at 387 nm is predominant, and other emissions due to defects in ZnO and MEH-PPV exciton emission are weaker. With increased applied voltage, the number of carriers injected into MEH-PPV increases and that trapped by ZnO defects also increases. Thus, three emissions appeared at high voltage: emission from ZnO, exciton emission of MEH-PPV and the defect emissions of ZnO [9].

When we deposited 0.8 nm LiF film on the MEH-PPV layers of the devices by thermal evaporation, the electroluminescence of ZnO at 387 nm was enhanced, and the emission of MEH-PPV was feeble. LiF is a material of interfacial modification of cathodes in organic electroluminescent device [17]. It is beneficial to the transport of electrons, which can increase the probability of electrons tunneled into the ZnO. More electrons were tunneled into the conduction band of the ZnO nanorods, and more electron–hole recombination existed in ZnO nanorods. Therefore, the recombination area is in ZnO nanorods, and the emission of MEH-PPV is very weak. The LiF film can separate the MEH-PPV layer and Al films. In addition, the presence of LiF film underneath Al electrode prevents the diffusion of Al atoms into MEH-PPV. The diffusion coefficient of Al can be 10^–18^ cm^2^/s at room temperature. If the Al atoms diffuse into the MEH-PPV layer, it will become easier for the holes to tunnel to the Al electrode and the electroluminescence will be bad. Fortunately, the LiF film can solve the problem and make the UV electroluminescence brighter. Comparatively from the normalized EL, we can find that using AZO as electrode and adding LiF film are positive for the UV electroluminescence.

Figure 5a,b show the I–V measurements and EL power of the three devices with different substrates and LiF layers. Clearly, the turn-on voltage of devices B and C are lower than that of device AII. The EL power of the devices B and C is higher than that of device AII. It is known that AZO has a larger work function relative to ITO. Therefore, the energy barrier between the valence band of ZnO and AZO should be small. Consequently, the hole injected into ZnO was augmented. This resulted in a decrease in the turn-on voltage of device B in comparison with device AII. The presence of LiF film at the interface layer between Al electrode and MEH-PPV is beneficial to the transport of electrons. When applying the same voltage to the device having LiF film, more electrons were tunneled into the conduction band of ZnO nanorods, unlike the device without LiF film. This caused more electron–hole recombination in ZnO nanorods layer. Thus, device C possesses lower turn-on voltage and enhanced EL power relative to device B. The EL power of device C increases fivefold relative to device AII. According to the results of electroluminescence, the UV emitted at 387 nm from device C is highly augmented relative to the emissions of other devices. This result reflects that the UV-electroluminescence performance of ZnO nanorods remarkably improved by fabricating the device with LiF film and AZO electrode. Previous published works explained the mechanism of UV electroluminescence as follows [9]. Negative carriers (electrons) are injected from the Al electrode into MEH-PPV; at the same time, positive carriers (holes) are injected from ITO (or AZO) electrode into the ZnO nanorods. Comparing the carriers’ concentration and carrier mobility of MEH-PPV with ZnO, MEH-PPV is considered as an insulator. When holes reach the interlayer between ZnO nanorods and MEH-PPV, they must accumulate at the interface. This accumulation arises due to the energy barrier between the valence band of ZnO and the highest occupied molecular orbital (HOMO) of MEH-PPV, which is equal to 2.3 eV. Accordingly, when increasing the applied voltage, the band of MEH-PPV will bend and a carrier tunneling operation becomes available. Electrons in the Al electrode tunnel through the MEH-PPV layer directly into the ZnO nanorods. These electrons recombine with the accumulated holes and produce the emission of 387 nm. The LiF film is beneficial to the electron injection. Under the same bias, the devices with LiF have a larger probability of electron tunneling than those without LiF. Thus, the turn-on voltage of the devices with LiF can be lower than those without LiF, and the EL power can be several times higher than those without LiF. 

## 5. Conclusions

In this work, the distribution of an electric field in the devices discussed above was analyzed by changing the thickness of MEH-PPV layer. The electric field on ZnO increases and then the hole injected becomes easy; the electrons tunnel easily through thinner MEH-PPV to ZnO. Therefore, carriers will prefer to recombine inside the ZnO layer, and the emission of ZnO is predominant under DC bias. It was shown that using AZO as an electrode and adding suitable thickness of LiF can strongly improve the near ultraviolet EL of the heterostructure of ZnO nanorods/MEH-PPV and decrease its turn-on voltage. This is because ITO has a lower work function compared to AZO. In such cases, there is a shrinkage in the energy barrier between the Fermi level of AZO and the valence band of ZnO, and the holes easily inject into ZnO. LiF film is beneficial to the injection of electron. Under the same bias, the devices with LiF have a larger probability of tunneling electrons than those without LiF. Thus, the LiF layer can obviously increase the EL power of the devices and decrease their turn-on voltage.
